# Acetaminophen is associated with improved survival in critically ill lung cancer patients: A propensity score-matched cohort study

**DOI:** 10.7150/ijms.122435

**Published:** 2026-01-14

**Authors:** Chen Chen, Weijia Zeng, Yunyi Li, Zhihui Yang, Xue He

**Affiliations:** 1 Department of Thoracic Surgery, The Second Xiangya Hospital of Central South University, Changsha, Hunan 410011, P.R. China; 2 Hunan Key Laboratory of Early Diagnosis and Precise Treatment of Lung Cancer, The Second Xiangya Hospital of Central South University, Changsha, Hunan 410011, P.R. China; 3 Department of Thoracic Intensive Care Unit, The Second Xiangya Hospital of Central South University, Changsha, Hunan 410011, P.R. China

**Keywords:** acetaminophen, mortality, critical patients, primary lung cancer, MIMIC-IV

## Abstract

**Background:** Acetaminophen is widely used in intensive care units, yet its impact on mortality among critically ill patients with primary lung cancer remains unclear. Given the high disease burden and potential immunomodulatory effects of acetaminophen, robust evidence is needed to clarify its prognostic relevance in this population.

**Methods:** We conducted a retrospective cohort study using the MIMIC-IV v2.2 database, including 1,127 critically ill patients with primary lung cancer. Baseline variables comprised demographics, comorbidities, illness severity scores (SOFA, APSIII, SAPSII, OASIS), and laboratory parameters. To minimize confounding, propensity score matching was applied.

**Results:** A total of 1,127 critically ill patients with primary lung cancer were included, of whom 403 received acetaminophen. The 28-day mortality rate was 22.0% in the acetaminophen group compared to 37.5% in the non-acetaminophen group. After adjusting for baseline differences using inverse probability of treatment weighting (IPTW), acetaminophen exposure was associated with a significantly lower risk of 28-day mortality (HR=0.75, 95% CI: 0.60-0.93, *p*=0.015). In addition to 28-day mortality, acetaminophen use was consistently associated with reduced risks of ICU mortality, in-hospital mortality, 30-day mortality, 90-day mortality, and 365-day mortality. Subgroup analyses identified patients aged ≥65 years and those with a SOFA score ≥3 as particularly noteworthy subgroups.

**Conclusion:** Acetaminophen use was associated with significantly reduced short- and long-term mortality in critically ill patients with primary lung cancer. These findings suggest a potential survival benefit beyond its conventional symptomatic use and underscore the need for prospective studies to validate its therapeutic role in this high-risk population.

## Introduction

Lung cancer is recognized as the foremost cause of oncological mortality globally among both genders, constituting approximately 28% of all cancer-related fatalities. In 2012, the World Health Organization documented 1.82 million new instances of lung cancer alongside 1.59 million deaths attributable to this disease 1. A substantial proportion of patients with lung cancer experience severe comorbidities and complications that require admission to the intensive care unit (ICU). ICU admission in this population is associated with markedly elevated mortality rates 2.

Acetaminophen (paracetamol) is commonly used for pain and fever management in ICU patients due to its perceived safety and favorable side effect profile compared to non-steroidal anti-inflammatory drugs (NSAIDs) and opioids. Its utilization has been associated with a decreased risk of lung cancer development 3. A substantial cohort study within the National Health and Nutrition Examination Survey (NHANES) revealed that ibuprofen usage could diminish the mortality risk from lung cancer by 48%, a benefit not observed with acetaminophen 1. Conversely, in patients with advanced lung cancer, high-dose exposure to acetaminophen may diminish overall survival rates when combined with immune checkpoint inhibitors 4. The role of acetaminophen in the ICU setting remains controversial 5. While a systematic review suggested that acetaminophen offers no clear benefit for short-term outcomes in critically ill patients 6, other studies have reported a potential association between acetaminophen use and reduced mortality in this population 7. However, the relationship between acetaminophen exposure and mortality, specifically in critically ill patients with lung cancer, remains poorly defined, warranting further investigation.

This study aimed to investigate the association between acetaminophen use and in-hospital mortality among ICU patients with a history of primary lung cancer. To achieve this, we executed a retrospective cohort analysis in patients who were diagnosed with primary lung cancer and classified as critically ill, utilizing data from the Medical Information Market for Intensive Care (MIMIC)-IV version 2.2. Our findings may help clarify the clinical value of acetaminophen in this high-risk population and guide evidence-based analgesic strategies.

## Methods

### Data sources and setting

The cohorts of patients analyzed in this investigation were derived from the MIMIC-IV database. Version 2.2 of MIMIC-IV comprises an extensive clinical dataset that includes detailed, high-caliber data on patients admitted to the Intensive Care Units at Beth Israel Deaconess Medical Center from 2008 to 2019. Access to this database was secured (certification number: 53984355). The study was conducted and reported following the Strengthening the Reporting of Observational Studies in Epidemiology (STROBE) guidelines.

### Study population

The study cohort comprised all individuals within the MIMIC-IV database, with inclusion criteria focusing on adults aged 18 years or older diagnosed with primary lung cancer. The study specifically included cases of malignant neoplasm of the bronchus and lung as well as malignant carcinoid tumors of the bronchus and lung, while excluding secondary malignant neoplasms of the lung. Patients were selected based on the International Classification of Diseases, Ninth Revision (ICD-9) and Tenth Revision (ICD-10) codes, including '1623', '1624', '1625', '1628', '1629', '20921', '2312', 'C34', 'C341', 'C3410', 'C3411', 'C3412', 'C342', 'C343', 'C3430', 'C3431', 'C3432', 'C348', 'C3480', 'C3481', 'C3482', 'C349', 'C3490', 'C3491', 'C3492', 'D022', 'D0220', 'D0221', 'D0222'. In instances of multiple ICU admissions, only the initial ICU admission was considered for analysis.

### Acetaminophen exposure

Exposure to acetaminophen was characterized by the administration of a prescription containing acetaminophen within 48 hours following hospital admission, with data being extracted from the MIMIC-IV database. We now state that a 48-hour window captures early administration at ICU entry and aligns with prior critical-care studies evaluating acetaminophen exposure and outcomes 7-9.

### Covariates

The analysis incorporated a comprehensive set of covariates: heart rate (HR), mean arterial pressure (MAP), oxygen saturation (SpO2), white blood cell count (WBC), hemoglobin levels, platelet count, blood urea nitrogen (BUN), creatinine levels, and several severity scoring systems such as the Acute Physiology Score (APS) III, the Simplified Acute Physiology Score (SAPS) II, the Sequential Organ Failure Assessment (SOFA) score, and the Oxford Acute Severity of Illness Score (OASIS). Additionally, comorbidities were meticulously cataloged, including myocardial infarction, congestive heart failure, peripheral vascular disease, cerebrovascular disease, chronic pulmonary disease, mild and severe liver disease, diabetes, paraplegia, and renal disease, alongside the Charlson Comorbidity Index, acetaminophen usage, systemic corticosteroid usage, and opioids usage. Demographic details acquired at the time of hospital admission registration encompassed age, sex, race, and admission type.

### Outcomes

The principal outcome measure was mortality within 28 days. Secondary outcome metrics encompassed mortality rates within the ICU, during the hospital stay, as well as at 90 days and 365 days post-admission.

### Statistical analysis

Multivariable Cox regression analyses were utilized to explore the independent association between acetaminophen exposure and 28-day mortality, employing an extended Cox model approach to adjust for various covariates 10. Survival outcomes were illustrated using Kaplan-Meier curves and assessed through log-rank tests. Subgroup analyses were conducted, segmented by pertinent covariates. A comprehensive descriptive analysis was performed on all study participants, with categorical variables presented as percentages and continuous variables as means with standard deviations or medians with interquartile ranges, depending on the distribution of the data. Comparative analyses of variables utilized chi-square tests for categorical data and One-Way ANOVA or Kruskal-Wallis tests for continuous data, based on the normality of the distribution. To balance the baseline characteristics between the groups, we used the PSM method, which was performed in our study by a 1:1 nearest neighbor matching algorithm with a caliper width of 0.2. The variables shown in Table 1 were all selected to generate the propensity score, and the standardized mean difference (SMD) was used to examine the degree of PSM. Less than 0.1 was considered an acceptable threshold. The primary outcome was further verified using inverse probability of treatment weighted (IPTW), which was created using the estimated propensity scores as weights. These analyses were executed using statistical software packages R version 3.3.2 and Free Statistics software version 1.9. Statistical significance was determined by a two-tailed test with a *p*-value threshold of less than 0.05.

## Results

### Study participants

**Figure 1:**
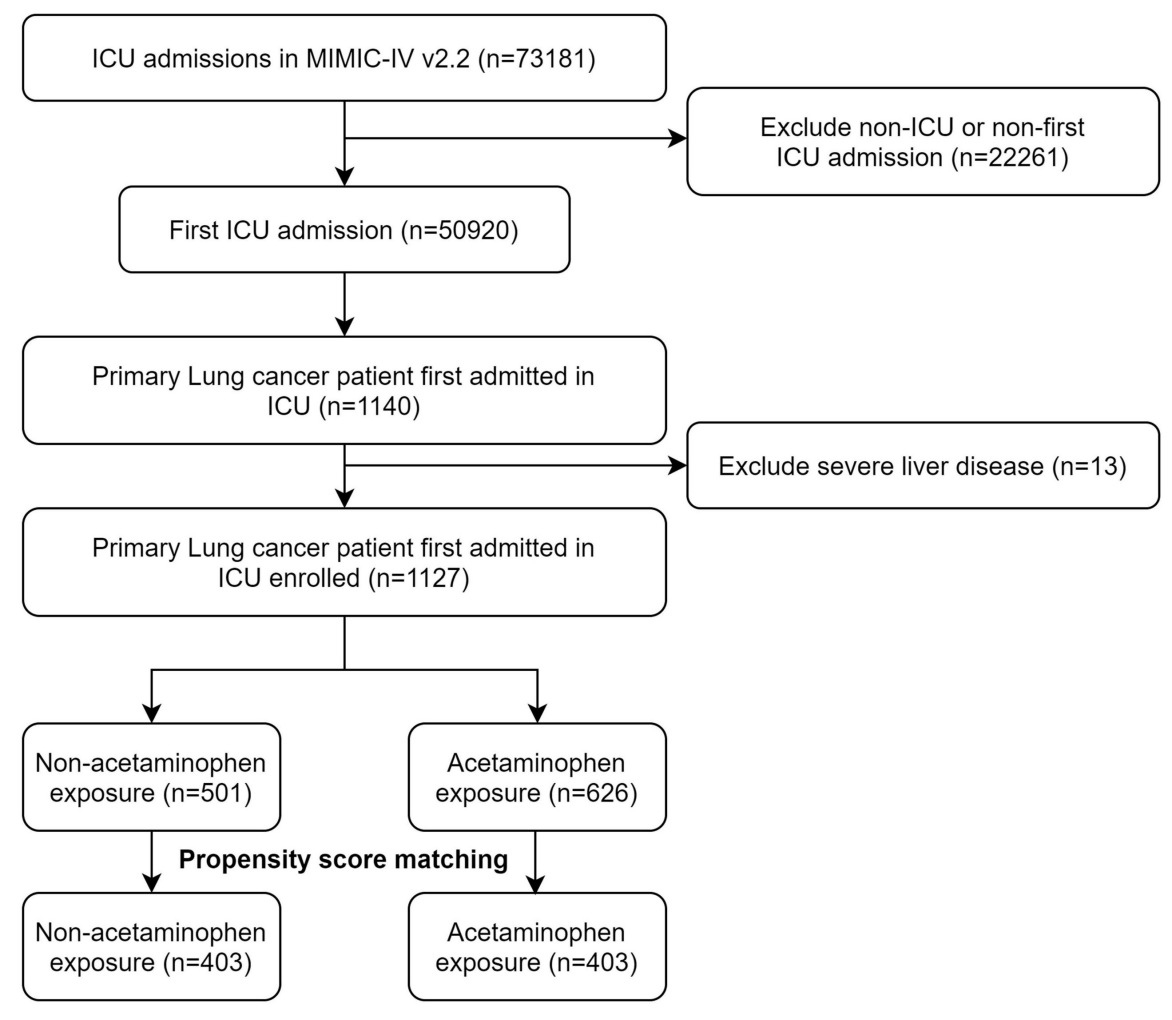
The flow chart of the study.

Within the confines of the MIMIC-IV database, we identified 1,127 critically ill patients diagnosed with primary lung cancer who met the study criteria (Figure 1). The dataset exhibited a scarcity of missing data, with less than 2% of relevant variables absent. The foundational characteristics and outcomes of these patients are delineated in Table 1. The average age within this cohort was approximately 69.6 years, with a standard deviation of 10.9 years, and 584 (51.0%) of the participants were male. Before PSM, there were minor imbalances in admission type and several severity scores, including SOFA and APS III. After PSM, no significant differences were observed between the acetaminophen and non-acetaminophen groups with respect to age (mean ± SD, 69.3 ± 11.2 vs 69.6 ± 11.4 years), sex (female, 49.8% vs 50.6%), race (White, 70.6% vs 71.2%), comorbidities (including myocardial infarction, congestive heart failure, diabetes), or severity of illness scores (SOFA, SAPS II, APS III, and OASIS). Laboratory tests and vital signs also showed comparable distributions after matching (Table 1).

### Primary outcome

**Figure 2:**
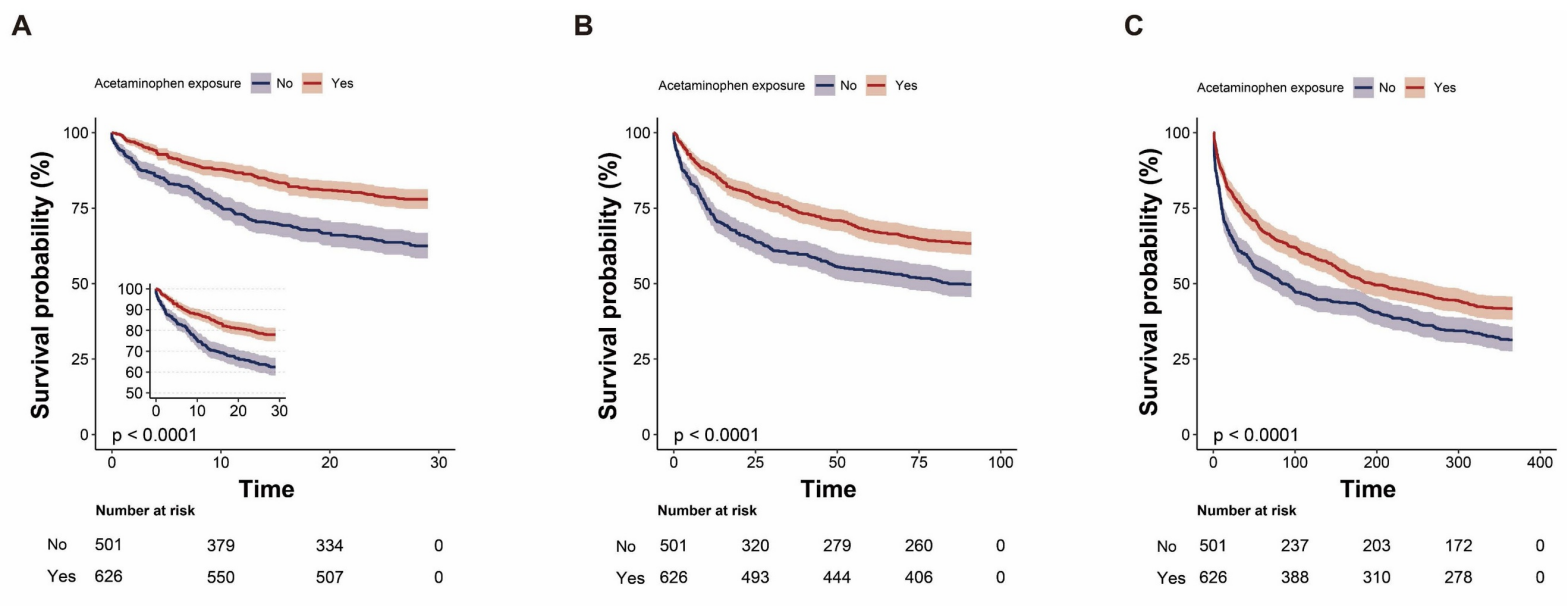
Kaplan-Meier survival curves comparing 28-day (A), 90-day (B), and 365-day (C) survival probabilities between the acetaminophen and non-acetaminophen groups for critically ill patients with primary lung cancer.

Before matching, acetaminophen exposure was associated with significantly lower 28-day mortality (22.0% vs 37.5%; p < .001). After PSM, this difference persisted (26.6% vs 33.3%; p = .038) (Table 2). In the unmatched cohort, acetaminophen exposure was associated with a significantly reduced risk of 28-day mortality (crude HR, 0.53; 95% CI, 0.42-0.66; p < .001). This association remained statistically significant across multivariable Cox regression (adjusted HR 0.68, 95% CI 0.54-0.87; p = .002), propensity score-matched Cox models (HR 0.76, 95% CI 0.59-0.98; p = .032), propensity score-adjusted models (HR 0.72, 95% CI 0.57-0.90; p = .004), and inverse probability-weighted (IPTW) models (HR 0.75, 95% CI 0.60-0.93; p = .015) (Table 3). The Kaplan-Meier survival curve further corroborated those patients who received acetaminophen exhibited a lower 28-day mortality rate (Log-rank test: *p* < 0.0001; Figure 2A).

### Secondary outcome

In-ICU mortality and in-hospital mortality were also lower in the acetaminophen group after matching (in-ICU: 9.4% vs. 14.4%, p = 0.030; in-hospital: 17.1% vs. 23.8%, p = 0.018). However, differences in 90-day and 365-day mortality were no longer statistically significant after matching (p = 0.119 and p = 0.712, respectively, Table 2). After adjusting for covariates, acetaminophen exposure remained independently associated with lower in-ICU mortality (adjusted HR 0.55, 95% CI 0.38-0.81; p = .003), in-hospital mortality (HR 0.60, 95% CI 0.45-0.80; p = .001), 90-day mortality (HR 0.79, 95% CI 0.65-0.95; p = .015), and 365-day mortality (HR 0.84, 95% CI 0.72-0.99; p = .033) (Table 4). The Kaplan-Meier survival curve further corroborated those patients who received acetaminophen exhibited a lower 90-day and 365-day mortality rate (Log-rank test: *p* < 0.0001; Figure 2B-C).

### Sensitivity analyses

The association between acetaminophen exposure and reduced 28-day mortality remained robust in sensitivity analyses restricted to patients without liver disease (adjusted HR 0.65, 95% CI 0.51-0.83; p = .001) and those who survived at least 24 hours post-ICU admission (adjusted HR 0.73, 95% CI 0.57-0.94; p = .013) (Table 5).

### Subgroup analysis

**Figure 3:**
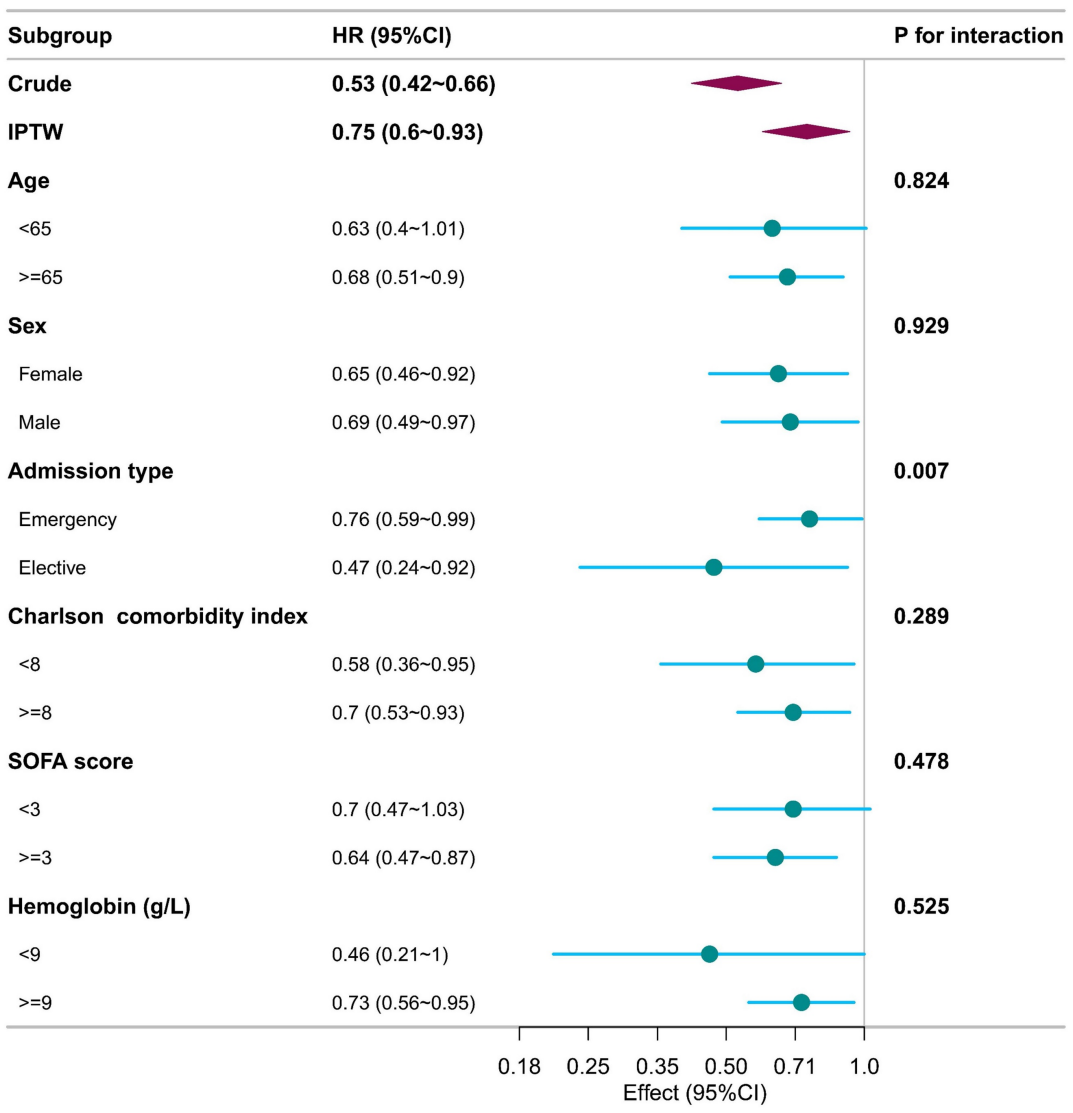
Subgroup analysis illustrates the relationship between acetaminophen use and 28-day mortality, with each subgroup analysis adjusting for all confounders listed in Table 1.

Subgroup analyses, conducted with respect to various confounders, consistently demonstrated that patients exposed to acetaminophen experienced lower 28-day mortality rates across all subgroups when compared to those who did not receive acetaminophen (Figure 3). The association between acetaminophen exposure and reduced 28-day mortality appeared more consistent and robust among patients aged 65 years or older and those with a SOFA score of 3 or higher. Nonetheless, notable interactions were identified between the type of admission and acetaminophen exposure, indicating a differential impact of acetaminophen based on the admission criteria (*p* for interaction < 0.05). As systemic corticosteroid and opioid use are common in the ICU, we performed subgroup analyses. Acetaminophen exposure was associated with lower 28-day mortality in both corticosteroid-exposed and nonexposed subgroups, as well as in the subgroup without opioid use (Supplemental Figure 1).

## Discussion

In this large retrospective cohort study, critically ill patients with primary lung cancer who received acetaminophen demonstrated significantly lower 28-day mortality compared to those who did not receive the drug. This association remained robust across multiple analytical approaches, including IPTW and propensity score matching. Notably, acetaminophen use was also associated with improved survival across both short-term and long-term timeframes. The observed survival benefit persisted across key predefined subgroups stratified by age, sex, admission type, Charlson Comorbidity Index, SOFA score, and hemoglobin levels, suggesting a consistent and potentially generalizable effect in this high-risk population.

Acetaminophen, widely used for its analgesic and antipyretic effects, has increasingly been associated with clinical outcomes such as mortality, acute kidney injury, and delirium in critically ill patients 9, 11, 12. A large multicenter cohort study from Australia involving 15,818 ICU patients and over 690,000 temperature measurements found that 64% of patients received at least 1 gram of paracetamol. Paracetamol use was associated with significantly lower in-hospital mortality (10% vs. 20%, p < 0.001), and a higher proportion of survivors had received paracetamol (66% vs. 46%, p < 0.001). This association remained consistent across both medical and surgical ICU subgroups 7. Similarly, a U.S. retrospective analysis reported that acetaminophen use in adult patients with sepsis was linked to reduced mortality and more ventilator-free days, though the sample size was limited 13.

However, other studies have raised questions about its clinical benefit. A systematic review suggested that paracetamol, while relatively safe, may not improve short-term outcomes in ICU patients 6. The ASTER randomized clinical trial found that intravenous acetaminophen did not significantly increase the number of days alive and free of organ support in patients with sepsis, although it was associated with lower SOFA scores and a reduced incidence of ARDS 14. Likewise, the HEAT trial showed that antipyretic treatment with acetaminophen in febrile ICU patients with suspected infection did not significantly affect ICU-free days or mortality, prompting a reevaluation of fever management in critical illness 15.

Aligning with these findings, our study contributes new evidence by demonstrating that acetaminophen use in critically ill patients with primary lung cancer is associated with lower short- (28-day) and long-term (90-day and 365-day) mortality, even after adjusting for temperature. One possible protective mechanism may involve the reduction of cell-free hemoglobin-induced oxidative injury, as suggested in previous studies 16, 17. The primary focus of this study was short-term mortality (28-day), which is most directly influenced by acute physiologic derangements and ICU-level interventions captured in MIMIC-IV. Although 90-day and 365-day mortality were analyzed as secondary outcomes, interpretation of long-term survival as a primary endpoint would require information on post-discharge clinical course, oncologic treatments, and disease progression, which are not available in this database. Accordingly, long-term mortality findings should be viewed as supportive and exploratory.

Subgroup analyses demonstrated a consistent association between acetaminophen exposure and lower 28-day mortality, which was robust among patients aged 65 years or older, across both sexes, across Charlson comorbidity index categories, among all admission types, in patients with a SOFA score of 3 or higher, and in those with hemoglobin levels >9 g/dL. Notably, a significant interaction was observed for admission type, with a more pronounced association in the elective admission subgroup. The interaction by admission type may reflect differences in clinical context, as elective ICU admissions are more often perioperative patients in whom acetaminophen is incorporated into multimodal analgesia, whereas emergency admissions frequently involve greater physiologic instability or infection. Opioids and systemic corticosteroids are ubiquitous in ICU care and can bias outcomes, while opioid use has been linked to worse short- and long-term survival 18, 19. Given this, we conducted subgroup analyses: acetaminophen exposure was associated with lower 28-day mortality in both corticosteroid-exposed and non-exposed subgroups, and the association persisted in the subgroup without opioid use. The absence of a clear association among opioid users does not contradict this finding, as opioid use in the ICU often serves as a marker of greater pain burden or disease severity and may attenuate the observable effect of acetaminophen through confounding by indication. Accordingly, these subgroup findings should be interpreted cautiously as hypothesis-generating. Further prospective studies with detailed perioperative, pharmacologic, and post-discharge data are warranted to confirm these observations and clarify the underlying mechanisms.

In oncology settings, acetaminophen is widely used as a non-opioid analgesic and antipyretic, particularly for managing mild to moderate cancer-related pain and fever, often in combination with opioids for more severe symptoms. Its mechanism involves central cyclooxygenase inhibition, resulting in reduced prostaglandin synthesis. Compared to NSAIDs, it has minimal peripheral anti-inflammatory effects but can alleviate fever and inflammatory discomfort 4. Emerging epidemiological evidence suggests a possible role in cancer prevention. Several population-based studies from the United States, Asia, and Denmark have reported associations between regular acetaminophen use and a modest reduction in lung cancer risk 3, 20, 21. However, high-dose acetaminophen, particularly when used in combination with immune checkpoint inhibitors, may be detrimental in advanced lung cancer patients, potentially compromising survival 22. These contradictory findings underscore the need for population-specific analyses. Our study adds crucial real-world evidence supporting a survival benefit associated with acetaminophen exposure in critically ill lung cancer patients.

Preclinical studies support the potential protective mechanisms underlying this association. Boutaud et al. demonstrated that acetaminophen mitigates hemoprotein-induced lipid peroxidation and improves renal function in animal models of oxidative stress, such as rhabdomyolysis, highlighting its antioxidant properties 23. Additionally, high-dose acetaminophen has been shown to inhibit cancer stem cell activity by suppressing the STAT3 signaling pathway, offering another plausible oncologic mechanism 24. These mechanistic insights, coupled with our clinical findings, suggest that acetaminophen may play a broader therapeutic role in cancer-related critical illness than previously appreciated.

A notable strength of our research is its pioneering focus on the association between acetaminophen usage and mortality among critically ill patients with lung cancer, a demographic not previously explored in depth. To reduce confounding, we employed propensity score matching to achieve well-balanced baseline characteristics between exposure groups, enhancing the internal validity of our findings. However, the study has its limitations. Firstly, its retrospective nature introduces potential selection and ascertainment biases 25. Secondly, as a single-center study, its findings possess internal validity but lack demonstrated external validity, limiting the generalizability of the results. Although some confounding factors were not fully addressed, the considerable sample size and extensive efforts to control for confounding bolster the study's credibility. Third, because MIMIC-IV lacks structured fields for tumor histology and stage, we could not adjust for these factors. Prospective studies should determine whether acetaminophen's effects differ by histologic subtype or stage. In addition, detailed information regarding cumulative dose, route of administration, and clinical indication for acetaminophen use was not consistently available in structured fields of the MIMIC-IV database, precluding dose-response or indication-specific analyses. This limitation should be considered when interpreting the observed associations. Ultimately, a prospective, randomized, double-blind, placebo-controlled trial in critically ill patients with primary lung cancer is warranted to definitively establish efficacy and safety.

## Conclusion

This retrospective analysis of the MIMIC-IV database suggests that acetaminophen use is associated with reduced mortality in critically ill patients with primary lung cancer. These findings highlight the potential clinical benefit of acetaminophen in this high-risk population. However, prospective, multicenter randomized controlled trials are warranted to confirm this association and to clarify the therapeutic role of acetaminophen in oncologic critical care.

## Supplementary Material

Supplementary figure.

## Figures and Tables

**Table 1:** Baseline characteristics of the included patients in the MIMIC-IV database

	Before PSM				After PSM			
Variables	Total (n = 1127)	Non- acetaminophen exposure (n = 501)	Acetaminophen exposure (n = 626)	SMD	Total (n = 806)	Non- acetaminophen exposure (n = 403)	Acetaminophen exposure (n = 403)	SMD
Age, Mean ± SD	69.6 ± 10.9	69.8 ± 11.1	69.4 ± 10.8	< 0.1	69.3 ± 11.2	69.6 ± 11.4	69.1 ± 11.0	< 0.1
Sex, n (%)				< 0.1				< 0.1
Female	552 (49.0)	254 (50.7)	298 (47.6)		401 (49.8)	204 (50.6)	197 (48.9)	
Male	575 (51.0)	247 (49.3)	328 (52.4)		405 (50.2)	199 (49.4)	206 (51.1)	
Race, n (%)				< 0.1				< 0.1
White	807 (71.6)	351 (70.1)	456 (72.8)		569 (70.6)	287 (71.2)	282 (70)	
Other	320 (28.4)	150 (29.9)	170 (27.2)		237 (29.4)	116 (28.8)	121 (30)	
Admission type, n (%)				> 0.1				< 0.1
Emergency	794 (70.5)	383 (76.4)	411 (65.7)		600 (74.4)	299 (74.2)	301 (74.7)	
Elective	333 (29.5)	118 (23.6)	215 (34.3)		206 (25.6)	104 (25.8)	102 (25.3)	
Comorbidity								
Myocardial infarct, n (%)	124 (11.0)	66 (13.2)	58 (9.3)	> 0.1	90 (11.2)	47 (11.7)	43 (10.7)	< 0.1
Congestive heart failure, n (%)	186 (16.5)	92 (18.4)	94 (15)	< 0.1	145 (18.0)	75 (18.6)	70 (17.4)	< 0.1
Peripheral vascular disease, n (%)	112 (9.9)	50 (10)	62 (9.9)	< 0.1	84 (10.4)	42 (10.4)	42 (10.4)	< 0.1
Cerebrovascular disease, n (%)	133 (11.8)	42 (8.4)	91 (14.5)	> 0.1	76 (9.4)	37 (9.2)	39 (9.7)	< 0.1
Chronic pulmonary disease, n (%)	517 (45.9)	232 (46.3)	285 (45.5)	<0.1	371 (46.0)	187 (46.4)	184 (45.7)	< 0.1
Diabetes, n (%)	221 (19.6)	91 (18.2)	130 (20.8)	< 0.1	154 (19.1)	75 (18.6)	79 (19.6)	< 0.1
Renal disease, n (%)	160 (14.2)	82 (16.4)	78 (12.5)	< 0.1	117 (14.5)	57 (14.1)	60 (14.9)	< 0.1
Mild liver disease, n (%)	55 (4.9)	26 (5.2)	29 (4.6)	> 0.1	33 (4.1)	16 (4)	17 (4.2)	< 0.1
Charlson comorbidity index, Mean ± SD	9.3 ± 2.5	9.4 ± 2.5	9.2 ± 2.6	< 0.1	9.3 ± 2.4	9.2 ± 2.4	9.3 ± 2.4	< 0.1
Severity of illness								
SOFA score, Median (IQR)	3.0 (1.0, 4.0)	3.0 (1.0, 5.0)	2.0 (1.0, 4.0)	> 0.1	2.0 (1.0, 4.0)	3.0 (1.0, 4.0)	2.0 (1.0, 4.0)	< 0.1
APS III, Mean ± SD	44.9 ± 20.7	48.0 ± 22.5	42.4 ± 18.7	> 0.1	45.0 ± 19.5	45.0 ± 19.2	45.1 ± 19.8	< 0.1
SAPS II, Mean ± SD	39.5 ± 12.4	40.6 ± 13.4	38.6 ± 11.4	> 0.1	39.1 ± 12.1	39.4 ± 12.1	38.9 ± 12.1	< 0.1
OASIS, Mean ± SD	32.0 ± 8.6	33.3 ± 9.1	30.9 ± 8.1	> 0.1	32.3 ± 8.3	32.4 ± 8.6	32.3 ± 8.0	< 0.1
Vital signs								
Heart rate, Mean ± SD	108.7 ± 21.2	111.0 ± 21.6	106.9 ± 20.8	> 0.1	109.6 ± 20.8	109.5 ± 20.6	109.7 ± 21.1	< 0.1
MAP, Mean ± SD	59.4 ± 13.0	58.4 ± 14.1	60.2 ± 12.1	> 0.1	59.9 ± 12.9	59.6 ± 13.5	60.3 ± 12.4	< 0.1
SpO2, Mean ± SD	90.4 ± 5.7	89.8 ± 6.2	90.9 ± 5.3	> 0.1	90.3 ± 5.6	90.3 ± 5.3	90.3 ± 6.0	< 0.1
Temperature, Mean ± SD	37.3 ± 0.6	37.2 ± 0.6	37.3 ± 0.7	> 0.1	37.2 ± 0.6	37.2 ± 0.6	37.3 ± 0.6	< 0.1
Laboratory tests								
WBC (x10^9^/L), Mean ± SD	14.0 ± 8.8	14.2 ± 9.8	13.8 ± 8.0	< 0.1	13.9 ± 8.4	14.4 ± 8.9	13.5 ± 7.9	< 0.1
Platelets (x10^12^/L), Mean ± SD	252.6 ± 126.0	252.2 ± 129.0	252.9 ± 123.6	< 0.1	253.0 ± 125.6	257.1 ± 129.1	248.9 ± 122.0	< 0.1
Hemoglobin (g/L), Mean ± SD	10.3 ± 2.1	10.2 ± 2.0	10.4 ± 2.2	< 0.1	10.3 ± 2.1	10.3 ± 2.0	10.4 ± 2.2	< 0.1
BUN (mg/dL), Median (IQR)	19.0 (14.0, 27.0)	20.0 (14.0, 29.0)	19.0 (14.0, 26.0)	> 0.1	19.0 (14.0, 28.0)	19.0 (13.5, 28.0)	20.0 (14.0, 27.0)	< 0.1
Creatinine (mg/dL), Median (IQR)	0.8 (0.6, 1.2)	0.8 (0.6, 1.3)	0.9 (0.7, 1.2)	> 0.1	0.8 (0.6, 1.2)	0.8 (0.6, 1.2)	0.9 (0.7, 1.2)	< 0.1

SOFA score, sequential organ failure score; APS III, acute physiology score; SAPS III, simplified acute physiology score; OASIS, outcome and assessment information set

**Table 2:** Outcomes of the included patients

	Before PSM				After PSM			
Variables	Total (n = 1127)	Non-acetaminophen exposure (n = 501)	Acetaminophen exposure (n = 626)	*p* value	Total (n = 806)	Non-acetaminophen exposure (n = 403)	Acetaminophen exposure (n = 403)	*p* value
**Primary Outcome**								
28-day mortality	326 (28.9)	188 (37.5)	138 (22)	< 0.001	241 (29.9)	134 (33.3)	107 (26.6)	0.038
**Secondary Outcomes**								
In-ICU mortality	143 (12.7)	93 (18.6)	50 (8)	< 0.001	96 (11.9)	58 (14.4)	38 (9.4)	0.03
In-hospital mortality	231 (20.5)	142 (28.3)	89 (14.2)	< 0.001	165 (20.5)	96 (23.8)	69 (17.1)	0.018
90-day mortality	482 (42.8)	252 (50.3)	230 (36.7)	< 0.001	360 (44.7)	191 (47.4)	169 (41.9)	0.119
365-day mortality	709 (62.9)	344 (68.7)	365 (58.3)	< 0.001	523 (64.9)	264 (65.5)	259 (64.3)	0.712

**Table 3:** Association between acetaminophen exposure and 28-day mortality using PSM

Models	HR (95%CI)	*p* value
Unmatched crude	0.53 (0.42~0.66)	< 0.001
Multivariable adjusted^a^	0.68 (0.54~0.87)	0.002
Propensity Score matched^b^	0.76 (0.59~0.98)	0.032
Propensity Score adjusted^c^	0.72 (0.57~0.9)	0.004
Weighted IPTW^d^	0.75 (0.6~0.93)	0.015

a, hazard ratio from the multivariable cox proportional model adjusted for all covariates (Table 1). b, hazard ratio from a multivariate cox proportional hazards model with the same strata and covariates matched according to the propensity score. The analysis comprised 806 patients, 403 of whom were exposed to acetaminophen and 403 who were not. c, odds ratio from a multivariable logistic proportional hazards model with the same strata and covariates, with additional adjustment for the propensity score. d, Primary analysis with a hazard ratio from the multivariable cox proportional hazards model with the same strata and covariates with inverse probability weighting according to the propensity score.

**Table 4:** Secondary outcome analysis

Secondary outcomes	Crude coefficient (95% CI)	Crude p-value	Adjusted coefficient (95% CI)	Adjusted p-value
In-ICU mortality	0.4 (0.28~0.56)	< 0.001	0.55 (0.38~0.81)	0.003
In-hospital mortality	0.45 (0.35~0.59)	< 0.001	0.6 (0.45~0.8)	0.001
90-day mortality	0.63 (0.52~0.75)	< 0.001	0.79 (0.65~0.95)	0.015
365-day mortality	0.71 (0.61~0.82)	< 0.001	0.84 (0.72~0.99)	0.033

**Table 5:** Sensitivity analysis of the relationship between acetaminophen exposure and 28-day mortality

Sensitive analysis	Crude coefficient (95% CI)	Crude p-value	Adjusted coefficient (95% CI)	Adjusted p-value
Non-liver disease	0.51 (0.41~0.64)	< 0.001	0.65 (0.51~0.83)	0.001
Live time >=24h	0.59 (0.46~0.74)	< 0.001	0.73 (0.57~0.94)	0.013

## Abbreviations

APS: Acute Physiology Score
BUN: Blood Urea Nitrogen
HR: Heart Rate
ICD: International Classification of Diseases
ICU: Intensive Care Unit
IPTW: Inverse Probability of Treatment Weighted
NHANES: National Health and Nutrition Examination Survey
MAP: Mean Arterial Pressure
MIMIC: Medical Information Market for Intensive Care
SAPS: Simplified Acute Physiology Score
SMD: Standardized Mean Difference
SOFA: Sequential Organ Failure Assessment
SpO2: Oxygen Saturation
STROBE: Strengthening the Reporting of Observational Studies in Epidemiology
OASIS: Oxford Acute Severity of Illness Score
PSM: Propensity Score Matching
WBC: White Blood Cell Count
