# Supplementary material for: Acetaminophen is associated with improved survival in critically ill lung cancer patients: A propensity score-matched cohort study
Source: Int J Med Sci. 2026 Jan 14;23(2):636–45. doi: 10.7150/ijms.122435 (PMC12825139; doi:10.7150/ijms.122435)

Supplemental figure 1. Subgroup analysis illustrates the relationship between acetaminophen use and 28-day mortality, with each subgroup analysis adjusting for all confounders listed in Table 1.

Subgroup	Total	Event (%)	HR (95%CI)	P for interaction
**Opioids**				**0.743**
No	519	100 (19.3)	0.67 (0.51~0.89)	
Yes	107	38 (35.5)	0.92 (0.53~1.59)	
**Corticosteroids**				**0.923**
No	545	126 (23.1)	0.69 (0.54~0.89)	
Yes	81	12 (14.8)	0.3 (0.11~0.83)	

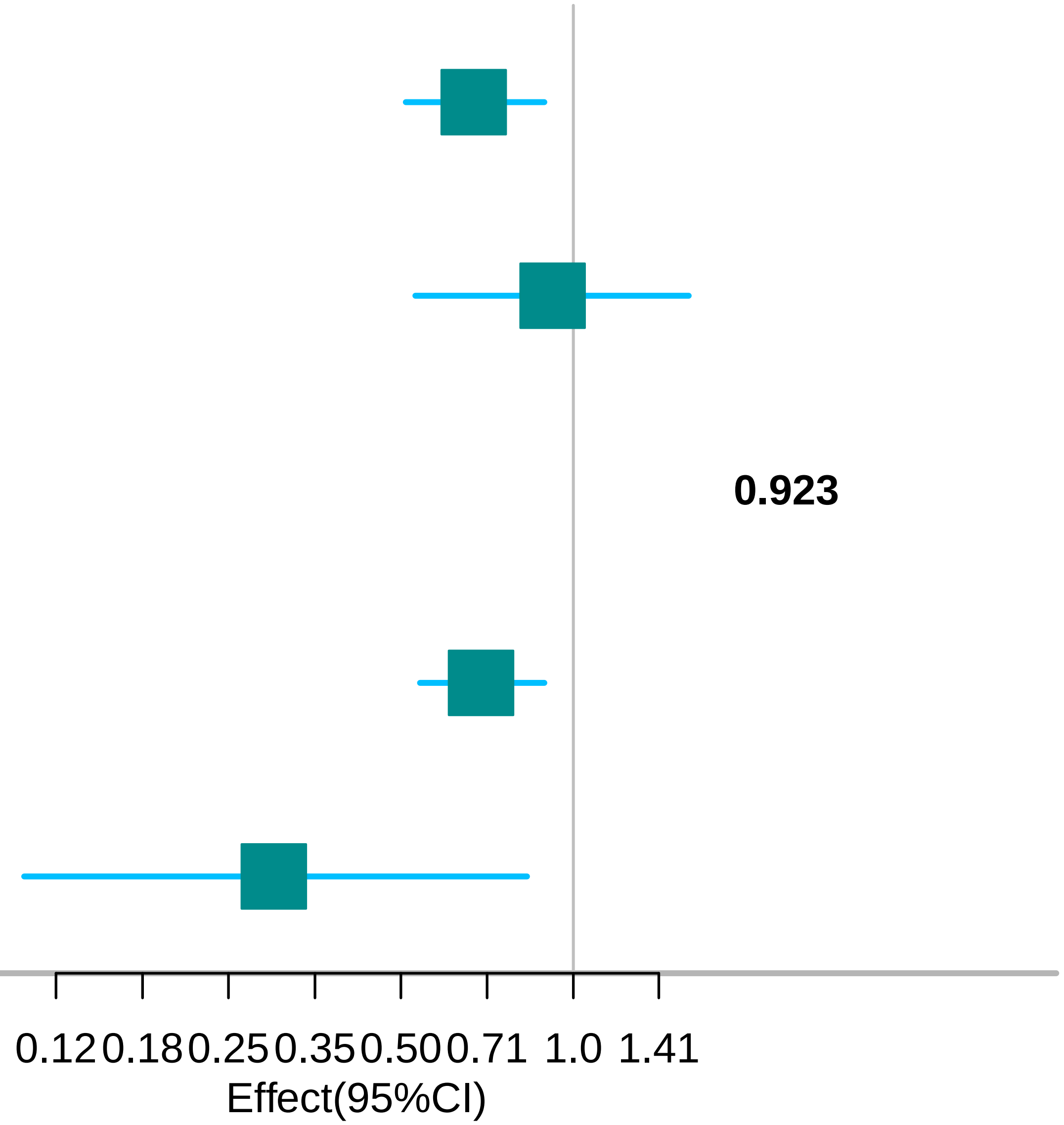

Supplement: Supplementary file 1 — Supplementary figure. [file ijmsv23p0636s1.pdf]
